# Cost-Effective In-House Negative Pressure Wound Therapy for Spinal Cord Injury Pressure Ulcer: A Case Report

**DOI:** 10.7759/cureus.97971

**Published:** 2025-11-27

**Authors:** Ajay Sarma Narayana Sarma, Heera Selsa

**Affiliations:** 1 Physical Medicine and Rehabilitation, Dr. Moopen's Medical College, Meppadi, IND

**Keywords:** cost-effective wound therapy, in-house negative pressure therapy, pressure ulcer, spinal cord injury, vacuum-assisted closure

## Abstract

Negative pressure wound therapy (NPWT) is an established method for enhancing the healing of severe pressure injuries (PIs), but its high cost often limits access in low-resource settings. We present a case of a 16-year-old male with cervical spinal cord injury (SCI) who developed a Grade IV sacral pressure ulcer that was successfully treated using an improvised, cost-effective, in-house NPWT system. At first, daily cleaning and moist-to-wet saline gauze dressings, applied twice daily for one month, led to partial improvement. Given limited progress and restricted resources, a novel wound management approach using an in-house NPWT system was adopted. The wound demonstrated dramatic improvement in surface area, depth, and exudate clearance after four weeks, with a total material cost of ₹2,300 (USD $26) per session. This case demonstrates the feasibility, safety, and effectiveness of locally adapted NPWT and provides valuable insight for wound care in similar settings.

## Introduction

Pressure injuries (PIs), also known as pressure ulcers or decubitus ulcers, result from prolonged pressure or shear forces that compromise skin and underlying tissues, typically over bony prominences. These injuries are particularly common among spinal cord injury (SCI) patients due to limited mobility and reduced sensation, posing serious risks such as infection, delayed wound healing, and diminished quality of life [1]. According to the National Pressure Injury Advisory Panel (NPIAP) staging system, PIs can be categorized into Stage 1 (intact skin with nonblanchable erythema), Stage 2 (partial-thickness skin loss involving the epidermis and dermis), Stage 3 (full-thickness skin loss with visible adipose (fat) and granulation tissue), Stage 4 (full-thickness skin and tissue loss with exposure), and Unstageable (depth unknown or obscured due to slough or eschar) [2]. Another grading system is the Pressure Ulcer Scale for Healing (PUSH), developed by the NPIAP, which grades pressure ulcers according to wound size, type of wound bed tissue, and amount of exudate [3,4]. The PUSH accurately differentiates between PIs that are healing and those that are not, and it offers a trustworthy way to track the healing of PIs over time. It is an evidence-based, clinically helpful measure for monitoring changes in PI status when applied on a weekly basis [5]. The treatment of advanced PIs, particularly Grade IV ulcers, remains a clinical challenge with significant economic implications. 

Negative pressure wound therapy (NPWT) has become an increasingly accepted method to promote healing in complex wounds. This technique works by applying controlled negative pressure to the wound bed, which helps decrease edema, increase local blood flow, remove infectious materials, and stimulate granulation tissue formation [1,6]. Despite demonstrated clinical benefits, the widespread use of commercially available NPWT systems is often hindered by high costs and resource limitations, especially in low- and middle-income healthcare environments [6].

To overcome these barriers, improvised in-house NPWT systems have been designed using readily available hospital supplies to mimic the effects of commercial devices at considerably lower costs. These affordable alternatives maintain the core therapeutic mechanisms of NPWT, offering a practical solution for individuals unable to access expensive equipment [6,7]. This case report details the successful application of a cost-effective, locally assembled NPWT system in a young SCI patient with a severe pressure ulcer, illustrating its efficacy and potential for broader adoption in resource-constrained settings [7].

## Case presentation

A 16-year-old male sustained a traumatic C4 SCI following a high-impact road traffic accident resulting in complete tetraplegia. After initial stabilization and surgical fixation, he was admitted for inpatient rehabilitation two weeks later. During his hospitalization, a Grade IV sacral pressure ulcer measuring 12 × 8 cm with a depth of 4 cm and a PUSH score of 16 was identified (Push Tool version 3.0, NPIAP) [4]. The ulcer was characterized by extensive full-thickness tissue loss with visible necrotic material and moderate exudate. The individual was unable to perform any activities of daily living (ADL) by self and was bowel and bladder incontinent. The patient’s medical history was otherwise unremarkable, with no known comorbidities that could impair wound healing. When used by a multidisciplinary team, the Spinal Cord Independence Measure (SCIM), which may be a helpful tool for evaluating how patients with spinal cord lesions perform on a daily basis, was measured to be 10 on 100, scoring only in the Respiration category [8]. A personalised diet that included high protein, micronutrient supply, iron supplementation, and adequate hydration was given for the entirety of the individual's stay for two months following a dietitian consultation and assessment. A daily diet chart, as well as a weekly nutritional screening and assessment, was done based on local hospital protocols to ensure adequate nutrient consumption to aid in faster healing. Repositioning every three hours when bed-bound, using the 30° tilt and the usage of pressure redistribution surfaces, positioning devices/pillows were used to offload pressure points, thereby reducing further incidences of PI's during hospital stay [9]. Initial standard wound care consisted of daily cleaning, debridement (if required), and moist-to-wet saline gauze dressings applied twice daily for one month, which led to a reduction in wound area to 10 × 6 cm, depth to 3 cm, and PUSH score to 14, as well as a decrease in necrotic tissue presence. A negative wound culture and sensitivity report was obtained following one month of normal saline (NS) dressings and debridement. Despite this improvement, healing progressed slowly with ongoing patient immobilization and a high risk of further tissue breakdown. Given the prohibitive cost of commercial NPWT devices, an in-house NPWT system was assembled and introduced as an adjunct to the existing wound care regimen. 

Method: in-house NPWT system application

Contents of the In-House NPWT System: (Total Cost of the System Components: ₹2,300 ($26USD))

**Figure 1:**
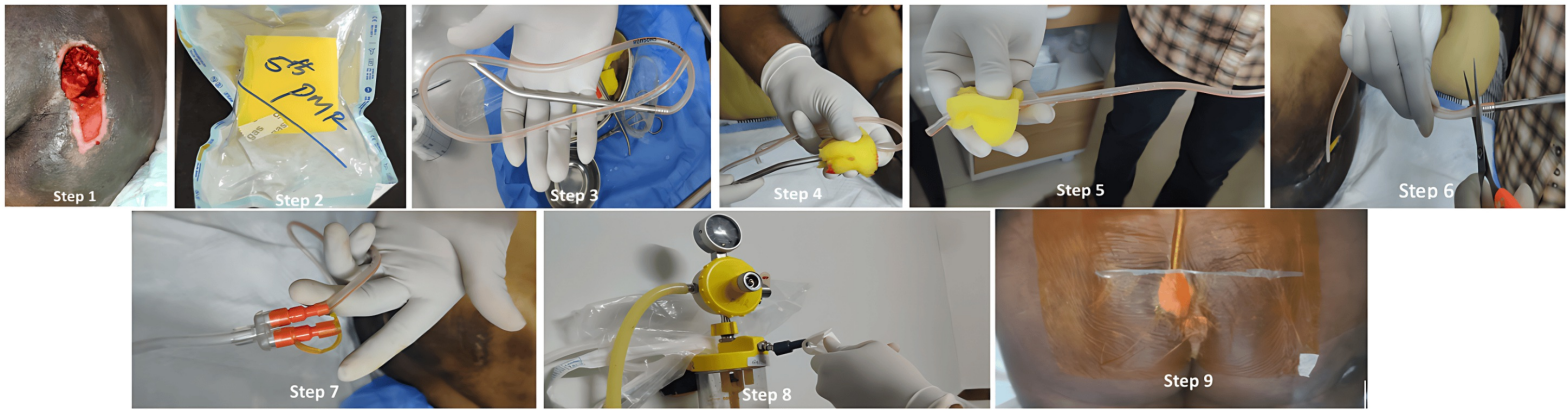
Steps of appplication of the in-house NPWT system Step 1: Measure wound size (length, width, and depth). Step 2: Cut foam to fit the wound cavity snugly. Steps 3, 4, and 5: Insert the drainage catheter through the cut foam. Step 6: The curved needle is cut off using a sterile blade/sterile scissors. Step 7: The cut foam with the drainage catheter in situ is placed in the wound. Step 8: Seal with an antimicrobial adhesive dressing and elastic bandage. Step 9: The free end of the catheter is connected to suction and checked for an airtight seal achieved. NPWT: negative pressure wound therapy

The in-house system used and its assembly have been adapted from published best practices on improvised NPWT, which include Dwivedi MK et al., Agarwal P et al., and Sifi N et al. [6,7,10]. The system comprised a wall-mounted suction device capable of providing continuous negative pressure of 80-125 mmHg, a Romo Vac Set® (Romsons Group Pvt Ltd, Agra, India), consisting of a kink-resistant connecting tubing, curved needle, and a bellows suction chamber, among which, only the tubing and curved needle were used. Furthermore, a sterilized polyurethane foam, cut to fit the wound, as well as an antimicrobial adhesive dressing to form an airtight seal, and an elastic bandage, for reinforcing the seal and tubing connections were used. The steps involved in assembling the in-house NPWT system are shown in Figure 1.

Outcome

The In-house NPWT system was applied weekly once for three days continuously [6,7]. The remaining four days were utilised to provide neuro-rehabilitation for the individual. NS dressings were also done in these four days, twice a day. Neuro-rehabilitation included occupational therapy and physiotherapy sessions, following which his SCIM progressed to 48 out of 100 with improvements seen in self-care and mobility (indoor, outdoor, and toilet). At the time of discharge, the individual was able to ambulate with a left Knee-Ankle-Foot Orthosis (KAFO) and a reciprocal walker with an endurance of 100 meters. Since there was a lack of voluntary control of voiding, he was provided a portable silicone catheter for long-term use. There was frequent constipation with significant defecatory difficulty and occasional incontinence, which was regularised with a personally catered bowel program [11].

A centimetre ruler was used to measure the PI's length and width, and a sterile cotton-tipped applicator was used to quantify the depth; it was introduced into the ulcer and marked at the deepest level [6]. Comparisons of length, breadth, area, depth, PUSH score, and rate of healing during initial presentation, after four weeks of conventional NS dressings, and after four weeks of in-house NS dressings + NPWT application are mentioned in Table 1. 

**Table 1:** Outcome following the addition of in-house NPWT dressing NS: normal saline, NPWT: negative pressure wound therapy

Measurements	During admission	After four weeks of NS dressing	After four weeks of NS + NPWT
Length	12 cm	10 cm	1 cm
Breadth	8 cm	6 cm	1 cm
Area	96 cm^2^	60 cm^2^	1 cm^2^
Depth	4 cm	3 cm	0.5 cm
PUSH Score	16	14	03
Rate of healing	Not applicable	1.2 cm^2^/day	2.0 cm^2^/day

**Figure 2:**
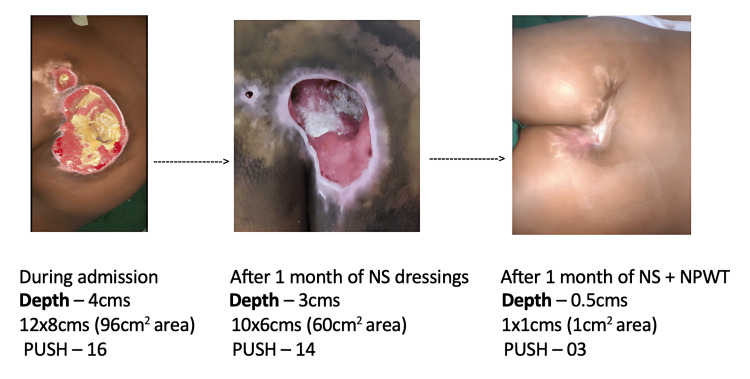
Wound progression after four weeks of conventional NS dressings and after four weeks of NS + in-house NPWT application NS: normal saline, NPWT: negative pressure wound therapy, PUSH: Pressure Ulcer Scale for Healing

Wound progression following one month of NS dressings and following one month of NS + NPWT system application is shown in Figure 2.

## Discussion

NPWT has become a cornerstone of modern wound management, particularly for complex and chronic ulcers in patients with SCI [1]. Its benefits, including rapid reduction in wound size, enhanced granulation tissue development, and decreased time to healing, have been consistently demonstrated across multiple studies [1,6]. Research shows that compared to conventional wound care, NPWT can significantly decrease hospitalization duration, reduce pain frequency, and improve quality of life, especially for patients with deep pressure ulcers such as those encountered in sacral locations [6,7].

In our patient, the improvised in-house NPWT system produced dramatic results, with near-complete closure of a Grade IV sacral ulcer in just four weeks. Clinical outcomes such as the reduction of wound area and depth, clearance of necrotic debris, and improvement in PUSH scores mirror published findings where low-cost or modified NPWT methods have proven as effective as commercial systems [6,7]. Several reports highlight that the main advantage of improvised NPWT devices lies in their accessibility, affordability, and adaptability to local resource limitations, allowing their use even in district hospitals and clinics without specialty wound care equipment [7,10].

Potential limitations of low-cost NPWT solutions include possible challenges in maintaining an airtight seal, variable negative pressure delivery, and restricted patient mobility/being bed-bound due to wall-connected suction devices [7,12]. However, studies suggest these challenges can be minimized through careful dressing technique and regular wound monitoring [7].

Unique aspects of this case include the young age of the patient, absence of comorbidities, and rapid tissue regeneration, which may have contributed to faster healing compared to older adult populations. Importantly, this approach aligns with recent international recommendations advocating the use of NPWT in PI management to reduce overall costs, shorten hospital stays, and improve wound closure rates [1,13].

In summary, the successful use of a locally assembled NPWT system in a severe SCI pressure ulcer supports broader adoption of such approaches in resource-constrained settings [6]. Further clinical research and protocol optimization could help standardize in-house NPWT use and expand advanced wound care access globally [7,13]. While the outcome is encouraging and supports the feasibility and safety of low-cost, improvised NPWT, strong conclusions on efficacy, generalizability, or best practice cannot be drawn on this basis alone [13].

## Conclusions

This case report demonstrates that a cost-effective, locally assembled in-house NPWT system can be a valuable addition to the management of severe Grade IV pressure ulcers in patients with SCI. The rapid and significant improvement observed in wound healing supports the use of improvised NPWT devices as a viable alternative to commercial systems in healthcare environments with limited resources.

Expanding adoption and structured evaluation of such approaches could improve access to advanced wound care and patient outcomes globally. Larger studies with higher subject numbers and comparative controls are needed to confirm the effectiveness, safety, and reproducibility of this approach. Until such data are available, our report serves primarily to document one successful, resourceful intervention and to encourage further research and clinical discussion.
